# *Tenebrio molitor* PGRP-LE Plays a Critical Role in Gut Antimicrobial Peptide Production in Response to *Escherichia coli*

**DOI:** 10.3389/fphys.2020.00320

**Published:** 2020-04-15

**Authors:** Maryam Keshavarz, Yong Hun Jo, Tariku Tesfaye Edosa, Yeon Soo Han

**Affiliations:** Department of Applied Biology, College of Agriculture and Life Sciences, Institute of Environmentally-Friendly Agriculture (IEFA), Chonnam National University, Gwangju, South Korea

**Keywords:** *Tenebrio molitor*, *TmPGRP-LE*, nuclear factor κB, expression patterns, antimicrobial peptides

## Abstract

Invading pathogens are recognized by peptidoglycan recognition proteins (PGRPs) that induce translocation of NF-κB transcription proteins and expression of robust antimicrobial peptides (AMPs). *Tenebrio molitor* PGRP-LE (*Tm*PGRP-LE) has been previously identified as a key sensor of *Listeria monocytogenes* infection. Here, we present that *TmPGRP-LE* is highly expressed in the gut of *T. molitor* larvae and 5-day-old adults in the absence of microbial infection. In response to *Escherichia coli* and *Candida albicans* infections, *TmPGRP-LE* mRNA levels are significantly upregulated in both the fat body and gut. Silencing of *TmPGRP-LE* by RNAi rendered *T. molitor* significantly more susceptible to challenge by *E. coli* infection and, to a lesser extent, *Staphylococcus aureus* and *C. albicans* infections. Reduction of *TmPGRP-LE* levels in the larval gut resulted in downregulation of eight AMP genes following exposure to *E. coli*, *S. aureus*, and *C. albicans*. However, the transcriptional levels of AMPs more rapidly reached a higher level in the ds*EGFP*-treated larval gut after challenge with *E. coli*, which may suggest that AMPs induction were more sensitive to *E. coli* than *S. aureus* and *C. albicans.* In addition, *TmPGRP-LE* RNAi following *E. coli* and *C. albicans* challenges had notable effects on *TmRelish*, *TmDorsal X1* isoform (*TmDorX1*), and *TmDorX2* expression level in the fat body and gut. Taken together, *TmPGRP-LE* acts as an important gut microbial sensor that induces AMPs via Imd activation in response to *E. coli*, whereas involvement of *TmPGRP-LE* in AMPs synthesize is barely perceptible in the hemocytes and fat body.

## Introduction

Invading infectious agents stimulate the innate immune system through complex intracellular pathways. To successfully combat invading pathogens, host immune cells express germline-encoded pathogen recognition receptors (PRRs) that recognize evolutionarily conserved molecular targets, known as pathogen-associated molecular patterns (PAMPs) (Kumar et al., 2011).

There is compelling evidence that innate immunity has ancient evolutionary origins in vertebrates and invertebrates. Phylogenetic studies of innate immune-related genes have revealed that mammals and insects share highly conserved generalized signaling components, which allows insects to be used as models for host-pathogen interaction studies (Ausubel, 2005). However, there are some considerable differences in the innate immune systems of vertebrates and invertebrates, including the mechanism of interaction between terminal molecules and receptors, the signal transduction pathways involved, and the effect of signaling pathway activation on the secretion or production of specific molecules (Sheehan et al., 2018). Mammalian Toll-like receptor (TLR) and tumor necrosis factor alpha (TNFα) signaling pathways are activated in response to invading microorganisms and this results in the translocation of NF-κB transcription factors to the nucleus, which stimulate the production of pro-inflammatory cytokines and chemokines, induce apoptosis, and connect innate and adaptive immunities (Ganesan et al., 2011; Ting and Bertrand, 2016). Insect NF-κB, Toll, and immune deficiency (Imd) signaling pathways, however, induce production of antimicrobial peptides (AMPs) (Lemaitre and Hoffmann, 2007).

The *Drosophila* Imd pathway (a homolog of the mammalian TNF pathway) is predominantly activated through the recognition of *meso*-diaminopimelic acid-containing peptidoglycans (DAP-type PGN) of gram-negative bacteria and some gram-positive bacteria (*Bacillus* and *Listeria* spp.) by peptidoglycan-recognition proteins (PGRPs) (Lemaitre and Hoffmann, 2007). The periplasmic murein (PGN) sacculus, which is composed of long glycan chains cross-linked by peptides, is the only cell surface component unique to bacteria, and is common to both gram-positive and gram-negative bacteria (Vollmer and Bertsche, 2008).

PGRP family members are evolutionarily conserved components of the innate immune system, with variants in insects and mammals sharing one or more C-terminal PGRP domain (around 165 amino acids) that is homologous to bacteriophage type 2 amidases and T7 lysozymes (Kang et al., 1998; Dziarski, 2004). However, some PGRPs do not have amidase activity, seemingly due to a reduction in number of amino acid residues necessary for cleavage and degradation of PGN in the presence of zinc (Wang et al., 2019).

The 19-kDa PGRP protein was first discovered in the hemolymph and cuticle of the silkworm, *Bombyx mori* (Yoshida et al., 1996). Since then, more than 100 PGRP orthologs have been identified in vertebrate and invertebrate species, including *Trichoplusia ni* (moth) (Kang et al., 1998), *Mus musculus* (mouse) (Kang et al., 1998), *Rattus norvegicus* (Rat) (Rehman et al., 2001), *Drosophila melanogaster* (Werner et al., 2000, 2003), *Anopheles gambiae* (mosquito) (Christophides et al., 2002), different fishes (Jang et al., 2013; Sun et al., 2014; Sun and Sun, 2015; Zhang et al., 2016), and humans (Liu et al., 2001). However, PGRP genes are not present in plants and nematodes (Dziarski and Gupta, 2006).

PGRPs can be separated into three categories based on general structure and transcript size: short extracellular PGRPs (PGRP-S, molecular weight of 20–25 kDa), intermediate PGRPs (PGRP-I, 40–45 kDa), and long intracellular, extracellular, or transmembrane PGRPs (PGRP-L, up to 90 kDa) (Dziarski, 2004; Mao et al., 2019). Previous studies have revealed a relatively high number of PGRP genes in insects (e.g., 13 genes in *Drosophila*) compared to mammals (only four PGRP genes). PGRP members can be further categorized into two groups: catalytic PGRPs and sensor PGRPs (non-catalytic). The former shares a conserved three-amino-acid structure critical for hydrolyzing PGN through cleavage of the amide bond between MurNAc and L-Ala (Mellroth et al., 2003; Guan et al., 2004). The latter does not cleave PGN due to a missing cysteine residue, but members of this group can bind to PNG, inducing Toll and Imd signaling pathways that lead to the production of AMPs (Table 1; Guan and Mariuzza, 2007; Wang et al., 2019).

**TABLE 1:** Identified PGRPs in insects and human.

Species name	Protein name	PGRP type	Function
*Drosophila*	*Dm*PGRP-SA	PGRP-S	Lys-type PGN Sensor in Toll pathway (Michel et al., 2001)
	*Dm*PGRP-SB1		Amidase activity toward DAP-type PGN in the Imd pathway (Zaidman-Rémy et al., 2011)
	*Dm*PGRP-SB2		Predicted amidase
	*Dm*PGRP-SC1a		Amidase activity toward Lys-type PGN in the Imd and Toll pathways (Costechareyre et al., 2016)
	*Dm*PGRP-SC1b		Amidase activity toward Lys-type PGN in the Imd and Toll pathways (Costechareyre et al., 2016)
	*Dm*PGRP-SC2		Amidase activity toward DAP-type and Lys-type PGN in the Imd and Toll pathways (Costechareyre et al., 2016)
	*Dm*PGRP-SD		Sensor PGRP in the Imd pathway (Iatsenko et al., 2016)
	*Dm*PGRP-LA	PGRP-L	Non-catalytic PGRP with no binding to PGN (Gendrin et al., 2013)
	*Dm*PGRP-LB		Amidase activity (Dziarski and Gupta, 2018)
	*Dm*PGRP-LC		Sensor PGRP in the Imd pathway (Kurata, 2010)
	*Dm*PGRP-LD		Unknown function
	*Dm*PGRP-LE		Sensor PGRP in the Imd pathway (Kurata, 2010; Bosco-Drayon et al., 2012)
	*Dm*PGRP-LF		Non-catalytic PGRP with no binding to PGN (Basbous et al., 2011)
*Homo sapiens*	PGLYRP-1 (PGRP-S)	PGRP-S	Bactericidal activity (Lu et al., 2006; Wang et al., 2007)
	PGLYRP-3 (PGRP-Iα)	PGRP-I	
	PGLYRP-4 (PGRP-Iβ)		
	PGLYRP-2 (PGRP-L)	PGRP-L	Amidase activity (Zhang et al., 2005)
*Tenebrio molitor*	*Tm*PGRP-SA	PGRP-S	DAP-type and Lys-type PGN Sensor in the Toll pathway (Park et al., 2006)
	*Tm*PGRP-SC2		Amidase activity and DAP-type PGN receptor in the Toll pathway (Yu et al., 2010)
	*Tm*PGRP-LE	PGRP-L	Sensor PGRP in the Imd pathway and phenoloxidase (PO) (Tindwa et al., 2013)

In *D. melanogaster*, PGRP-LE (*Dm*PGRP-LE) is a long, secreted PGRP that acts as an intracellular and extracellular microbial sensor. *Dm*PGRP-LE recognizes the polymeric DAP-type PGN and tracheal cytotoxin (TCT, a monomeric DAP-type PGN) from gram-negative bacteria and *Listeria monocytogenes*. Binding of the ligand triggers independent activation of the Imd pathway and induces autophagy (Kaneko et al., 2006; Yano et al., 2008). Following *L. monocytogenes* challenge, stimulation of the Janus kinase-signal transducers and activators of transcription (JAK-STAT) pathway results in *Listericin* induction, which is cooperatively regulated by *Dm*PGRP-LE (Goto et al., 2010). After PGRP-LE binds to DAP-type PGN in the cytoplasm of immune cells, it interacts synergistically with PGRP-LC, which in-turn activates the receptor multimerization that is required for signal transduction in the Imd pathway. Moreover, overexpression of PGRP-LE and PGRP-LC leads to activation of the prophenoloxidase cascade (Takehana et al., 2004; Kurata, 2014). Exploration of the *D. melanogaster-Photorhabdus* model has highlighted the importance of *DmPGRP-LE* in the hemolymph of flies during immune response to *Photorhabdus luminescens* and *Photorhabdus asymbiotica*, but not to non-pathogenic *Escherichia coli* (Chevée et al., 2019). Interestingly, *PGRP-LE* knockdown in the pupae of the red flour beetle, *Tribolium castaneum*, has been shown to decrease the expression of *Defensin3* following *E. coli* challenge, suggesting that *TcPGRP-LE* has independent activity against gram-negative bacteria (Koyama et al., 2015).

In a related study, the immune significance of *Tenebrio molitor* PGRP-SA (*Tm*PGRP-SA) and *Tm*PGRP-SC2 proteins was demonstrated (Table 1; Yu et al., 2010). Our team has previously identified the *Tm*PGRP-LE protein (37.3 kDa) as a non-catalytic PGRP that is essential for larval survival upon *L. monocytogenes* infection (Tindwa et al., 2013). Taking into account the known biological differences in immune responses between *Tenebrio* and *Drosophila*, the focus of this study was to investigate the role of *Tm*PGRP-LE as a multifunctional component of the Imd pathway, specifically as a fine regulator of key immune-related genes (including AMP genes) in the fat body, gut, and hemocytes of *Tenebrio molitor* following challenges with *E. coli*, *Staphylococcus aureus*, or *Candida albicans*.

## Materials and Methods

### *T. molitor* Maintenance

Stocks were raised in an insectarium on an artificial diet at 27 ± 1°C and 60 ± 5% relative humidity in the dark, in accordance with a previous study (Keshavarz et al., 2019). Healthy, normal larvae at the 10th–12th instar (around 2.4 cm in length) were used for experiments (*n* = 20 per group).

### Gene Expression Analysis of *TmPGRP-LE* in the Larval *T. molitor* Tissues

Evidence of the constitutive expression of *TmPGRP-LE* during the developmental stages (Tindwa et al., 2013) prompted us to elucidate the tissue-specific pattern of *TmPGRP-LE* expression in the integument, fat body, hemocytes, gut, Malpighian tubules, ovary, and testes of larvae and adults.

Total RNA was extracted from all tissue samples using the LogSpin RNA isolation method with minor modifications (Yaffe et al., 2012). Briefly, the samples were homogenized in guanidine thiocyanate-based RNA lysis buffer mixed with 99% ethanol and transferred to silica spin columns (Bioneer, Korea, KA-0133-1). The resultant RNA was treated with DNase (Promega, M6101, United States) to eliminate the genomic DNA contamination for 15 min at 37°C. Preceding all steps each silica column was then washed twice with the supplied wash buffers using 3 M sodium acetate buffer and 80% ethanol and dried for 1 min. Total RNA was eluted with 30 μL of distilled water (Sigma, W4502-1L, United States). Next, 2 μg of total RNAs were converted to cDNA using the AccuPower^®^ RT PreMix (Bioneer, Korea) kit with an oligo-(dT) ^12–18^ primer according to the protocol recommended by the manufacturer. The synthesized cDNAs (1:20 dilution with DNase/RNase free water) processed for quantitative reverse-transcription PCR (qRT-PCR) using AccuPower^®^ 2X GreenStar qPCR Master Mix (Bioneer, Korea). Data was normalized to *T. molitor 60 S ribosomal protein L27a* (*TmL27a*) transcripts and values were calculated using the comparative C_T_ method (2^–ΔΔ*CT*^ method) (Schmittgen and Livak, 2008). Specific primers for qRT-PCR were designed using Primer 3.0 plus^1^ and are listed in Table 2. For detailed information, including PCR conditions, see our previous paper (Keshavarz et al., 2019).

**TABLE 2:** Sequences of primer pairs used in this study.

Primer	Sequence (5′–3′)
*Tm*PGRP-LE_qPCR_Fw	5′-CTTCGCTTGCGGAATGGCAGATTA-3′
*Tm*PGRP-LE_qPCR_Rv	5′-AACACACGCTCAAATCCTTTCCCG-3′
ds*Tm*PGRP-LE_Fw	5′-TAATACGACTCACTATAGGGAGGCAACGT
	AAATAAGGACGG-3′
ds*Tm*PGRP-LE_Rv	5′-TAATACGACTCACTATAGGGAGTAGGCGA
	TATCGTTCCACTTC-3′
dsEGFP_Fw	5′-TAATACGACTCACTATAGGGTACGTAAAC
	GGCCACAAGTTC-3′
dsEGFP_Rv	5′-TAATACGACTCACTATAGGGTTGCTCAGG
	TAGTGTTGTCG-3′
*Tm*Tenecin-1_Fw	5′-CAGCTGAAGAAATCGAACAAGG-3′
*Tm*Tenecin-1_Rv	5′-CAGACCCTCTTTCCGTTACAGT-3′
*Tm*Tenecin-2_Fw	5′-CAGCAAAACGGAGGATGGTC-3′
*Tm*Tenecin-2_Rv	5′-CGTTGAAATCGTGATCTTGTCC-3′
*Tm*Tenecin-3_Fw	5′-GATTTGCTTGATTCTGGTGGTC-3′
*Tm*Tenecin-3_Rv	5′-CTGATGGCCTCCTAAATGTCC-3′
*Tm*Tenecin-4_Fw	5′-GGACATTGAAGATCCAGGAAAG-3′
*Tm*Tenecin-4_Rv	5′-CGGTGTTCCTTATGTAGAGCTG-3′
*Tm*Defensin-1_Fw	5′-AAATCGAACAAGGCCAACAC-3′
*Tm*Defencin-1_Rv	5′-GCAAATGCAGACCCTCTTTC-3′
*Tm*Defensin-2_Fw	5′-GGGATGCCTCATGAAGATGTAG-3′
*Tm*Defensin-2_Rv	5′-CCAATGCAAACACATTCGTC-3′
*Tm*Coleoptericin-1_Fw	5′-GGACAGAATGGTGGATGGTC-3′
*Tm*Coleoptericin-1_Rv	5′-CTCCAACATTCCAGGTAGGC-3′
*Tm*Coleoptericin-2_Fw	5′-GGACGGTTCTGATCTTCTTGAT-3′
*Tm*Coleoptericin-2_Rv	5′-CAGCTGTTTGTTTGTTCTCGTC-3′
*Tm*Attacin-1a_Fw	5′-GAAACGAAATGGAAGGTGGA-3′
*Tm*Attacin-1a_Rv	5′-TGCTTCGGCAGACAATACAG-3′
*Tm*Attacin-1b_Fw	5′-GAGCTGTGAATGCAGGACAA-3′
*Tm*Attacin-1b_Rv	5′-CCCTCTGATGAAACCTCCAA-3′
*Tm*Attacin-2_Fw	5′-AACTGGGATATTCGCACGTC-3′
*Tm*Attacin-2_Rv	5′-CCCTCCGAAATGTCTGTTGT-3′
*Tm*Cecropin-2_Fw	5′-TACTAGCAGCGCCAAAACCT-3′
*Tm*Cecropin-2_Rv	5′-CTGGAACATTAGGCGGAGAA-3′
*Tm*Thaumatin-like protein-1_Fw	5′-CTCAAAGGACACGCAGGACT-3′
*Tm*Thaumatin-like protein-1_Rv	5′-ACTTTGAGCTTCTCGGGACA-3′
*Tm*Thaumatin-like protein-2_Fw	5′-CCGTCTGGCTAGGAGTTCTG-3′
*Tm*Thaumatin-like protein-2_Rv	5′-ACTCCTCCAGCTCCGTTACA-3′
*Tm*Relish_qPCR_Fw	5′-AGCGTCAAGTTGGAGCAGAT-3′
*Tm*Relish_qPCR_Rv	5′-GTCCGGACCTCATCAAGTGT-3′
*Tm*DorX1_qPCR_Fw	5′-AGCGTTGAGGTTTCGGTATG-3′
*Tm*DorX1_qPCR_Rv	5′-TCTTTGGTGACGCAAGACAC-3′
TmDorX2_qPCR_Fw	5′-ACACCCCCGAAATCACAAAC-3′
TmDorX2_qPCR_Rv	5′-TTTCAGAGCGCCAGGTTTTG-3′
*Tm*L27a_qPCR_Fw	5′-TCATCCTGAAGGCAAAGCTCCAGT-3′
*Tm*L27a_qPCR_Rv	5′-AGGTTGGTTAGGCAGGCACCTTTA-3′

*Underline indicates T7 promotor sequences.*

### Microbial Strains and Infection Experiments

The gram-negative bacterium *E. coli* (starin K12) and gram-positive bacterium *S. aureus* (strain RN4220) were cultured in Luria-Bertani (LB) broth, and the fungus *C. albicans* was cultivated in Sabouraud dextrose broth at 37°C overnight. Cells from overnight microbial cultures were concentrated by centrifugation at 3,500 rpm for 15 min at room temperature (∼25°C). The supernatant was discarded, after which the pellet was washed (three times) and resuspended in phosphate-buffered saline (PBS; pH 7.0). The cell concentration was determined by optical density (OD) measurement at 600 nm. Based on OD_600_ values, the microorganism suspensions were adjusted to 10^6^ cells/μL for *E. coli* and *S. aureus*, and 5 × 10^5^ cells/μL for *C. albicans*.

Microbial challenges were conducted on *T. molitor* larvae (10th–12th instar) by injecting 1 μL of *E. coli* (1 × 10^6^ cells/μL), *S. aureus* (1 × 10^6^ cells/μL), *C. albicans* (5 × 10^4^ cells/μL), or PBS control between the 3rd and 4th abdominal segment. For quantification of *TmPGRP-LE* mRNA expression, whole insects or dissected immune-related tissues (fat body, gut, and hemocytes) were collected at various time points (at 3, 6, 9, 12, and 24 h post-infection). Subsequently, total RNA extraction, cDNA synthesis, and qRT-PCR were performed as previously described.

### *TmPGRP-LE* Silencing

Double-stranded RNA for use in RNAi experiments were synthesized as previously described (Tindwa et al., 2013). Concisely, the PCR product (636 bp sequence) tailed with T7 promotor sequence of *TmPGRP-LE* was amplified using AccuPower^®^ Pfu PCR PreMix with forward (ds*TmPGRP-LE*_Fw) and reverse (ds*TmPGRP-LE*_Rv) primers under the following conditions: denaturation at 95°C for 2 min, followed by 30 cycles of denaturation at 95°C for 20 s, annealing at 56°C for 30 s, and extension at 72°C for 5 min (Table 2). The synthesized ds*TmPGRP-LE* was purified using an AccuPrep^®^ PCR Purification Kit (Bioneer, Korea). Subsequently, the purified product was used as a template to synthesize ds*TmPGRP-LE in vitro* using an EZ^TM^ T7 High Yield *in vitro* Transcription Kit (Enzynomics, Korea) as per the manufacturer’s instructions. Then it was precipitated with 5 M ammonium acetate and washed with 70, 80, and 99.9% ethanol sequentially. After drying at room temperature, the precipitate was resuspended in 30 μL distilled water (Sigma, W4502-1L, United States) to obtain the final product.

An additional dsRNA stock of *EGFP* (ds*EGFP*) was generated using the PCR product (546 bp sequence) of the *enhanced green fluorescent protein* (*EGFP*) gene derived from the plasmid EGFP-C1. ds*EGFP* was used as a negative control in subsequent RNAi experiments.

### Survival Experiments

A key question concerning *TmPGRP-LE* is its role in combatting larval infection; to address this question, we examined the viability of larvae exposed to microbes after silencing *TmPGRP-LE* expression. However, it is crucial to verify that merely *TmPGRP-LE* depletion do not affect the survival percent. To quantitatively address this question, we injected 1 μL (1 μg) of ds*TmPGRP-LE* into 10th–12th instar *T. molitor* larvae. The knockdown efficiency for the target gene (*TmPGRP-LE*) was measured on the 6th day post-treatment. After confirmation of silencing, dsRNA-injected larvae (*n* = 10 per group) were challenged with *E. coli* (1 × 10^6^ cells/μL), *S. aureus* (1 × 10^6^ cells/μL), or *C. albicans* (5 × 10^4^ cells/μL). PBS were injected into dsRNA-injected larvae as a control. Survivors were counted daily for a duration of 10 days. The experiments were repeated three times, with 10 larvae per group for each experiment.

### Effect of *TmPGRP-LE* Silencing on AMP Genes and NF-κB Genes Expression Post-Microbial Challenge

To understand the function of *TmPGRP-LE* in regulating AMP genes, we examined the gene expression profiles of 14 AMPs, namely *TmTenecin*-1 (*TmTene1*), *TmTenecin*-2 (*TmTene2*), *TmTenecin*-3 (*TmTene3*), *TmTenecin*-4 (*TmTene4*), *TmAttacin-1a* (*TmAtt1a*), *TmAttacin-1b* (*TmAtt1b*), *TmAttacin-2* (*TmAtt2*), *TmDefensin*-1 (*TmDef1*), *TmDefensin*-2 (*TmDef2*), *TmColeoptericin-1* (*TmCole1*), *TmColeoptericin-2* (*TmCole2*), *TmCecropin-2* (*TmCec2*), *TmThaumatin-like protein-1* (*TmTLP1*), and *TmThaumatin-like protein-2* (*TmTLP2*) in the *TmPGRP-LE*-silenced larvae after microbial challenges. In addition to AMP gene expression profiling, the mRNA levels of three previously identified transcription factors composed of *TmRelish*, *TmDorsal X1* isoform (*TmDorX1*), and *TmDorsal X2* isoform (*TmDorX2)* were also measured. ds*EGFP* was used as a negative control, and PBS served as a wound control. Knowledge of the immunological role of *TmPGRP-LE* in different tissues is valuable; thus, we dissected the fat body, gut, and hemocytes of experimental samples 24 h post-injection. Each experiment was independently repeated trice (*n* = 20 per group). Samples were processed for cDNA synthesis, and qRT-PCR analysis was conducted using AMP-specific primers (Table 2).

### Statistical Analysis

Three independent biological replicates were used for all experiments. Values were reported as mean ± SE. Differences between groups were analyzed using one-way statistical analysis of variance (ANOVA) and Tukey’s test; *p* < 0.05 were considered significant. The results for the mortality assay were analyzed using the Kaplan-Meier plot (log-rank Chi-square test) in Excel.^2^

## Results

### Tissue-Specific Expression Patterns of *TmPGRP-LE*

As an intracellular receptor, cytoplasmic *Dm*PGRP-LE was previously detected in hemocytes and Malpighian tubules. However, as an extracellular PGN receptor, it was previously detected in the fat body and hemocytes; in these tissues, *Dm*PGRP-LE can enhance *Dm*PGRP-LC-mediated recognition of PGN and activate the Imd signaling pathway (Takehana et al., 2004; Kaneko et al., 2006). Similar to other long PGRPs (excluding PGRP-LB), *Tm*PGRP-LE lacks a signal peptide. Thus, it is constitutively expressed in the cytoplasm during all developmental stages and acts as an intracellular scavenger of PGN (Tindwa et al., 2013).

**FIGURE 1:**
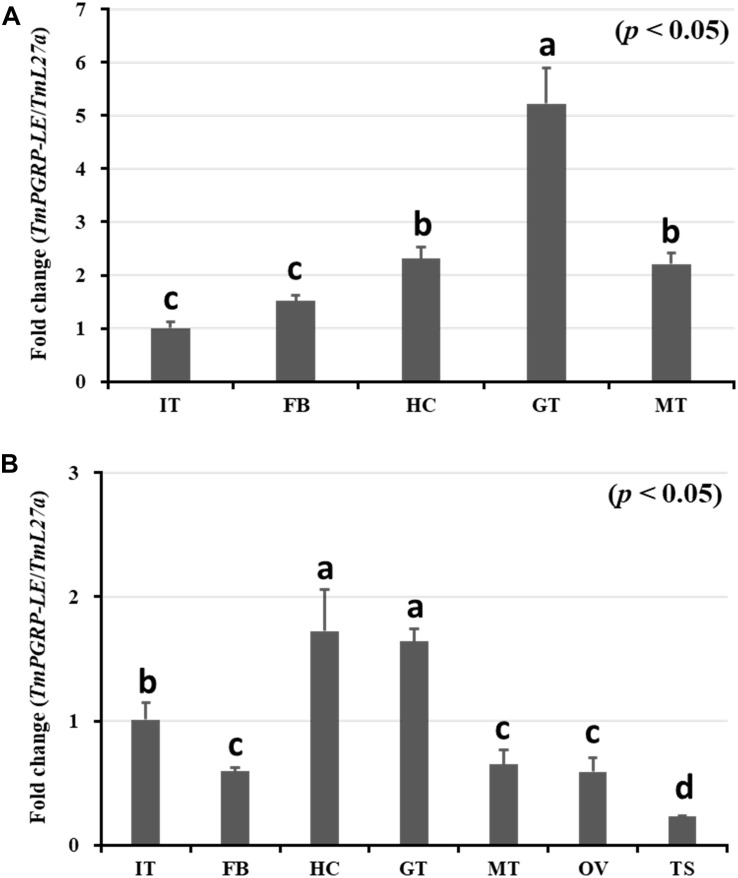
Quantitative determination of *TmPGRP-LE* expression in different tissues of late-instar *T. molitor* larvae **(A)** and 5-day-old adults **(B)** measured by qRT-PCR. The mRNA levels of *TmPGRP-LE* in the integument (IT), fat body (FB), hemocytes (HC), gut (GT), and Malpighian tubules (MT) are shown for late-instar larvae **(A)**. *TmPGRP-LE* transcripts in these tissues, as well as in the ovary (OV) and testis (TS), are shown for 5-day-old adults **(B)**. All measurements are depicted relative to the expression levels of *T. molitor 60S ribosomal protein L27a* (*TmL27a*) as an endogenous control. Each vertical bar represents mean ± SE (*n* = 20 per group). The significant differences between groups were determined using One-way ANOVA and Tukey’s multiple range test at 95% confidence level (*p* < 0.05). Bars in each graph with the same letter are not significantly different from each other.

In this study, we employed qRT-PCR to investigate the tissue distribution of *TmPGRP-LE* transcripts (Figure 1). *TmPGRP-LE* mRNA was detected in all *T. molitor* larval tissues, with the highest expression level observed in the gut, followed by that in hemocytes and Malpighian tubules, while the lowest mRNA quantities were found in the integument and fat body (Figure 1A). Similarly, there was high expression of *Tm*PGRP-LE in the gut and hemocytes of 5-day-old adults, with lower expression found in the integuments. The transcription of *TmPGRP-LE* was weakly detected in the fat body, Malpighian tubules, and ovary. The lowest expression of Tm*PGRP-LE* was found in the testis (Figure 1B).

### Induction Profiles of *TmPGRP-LE* Upon Microbial Challenge

**FIGURE 2:**
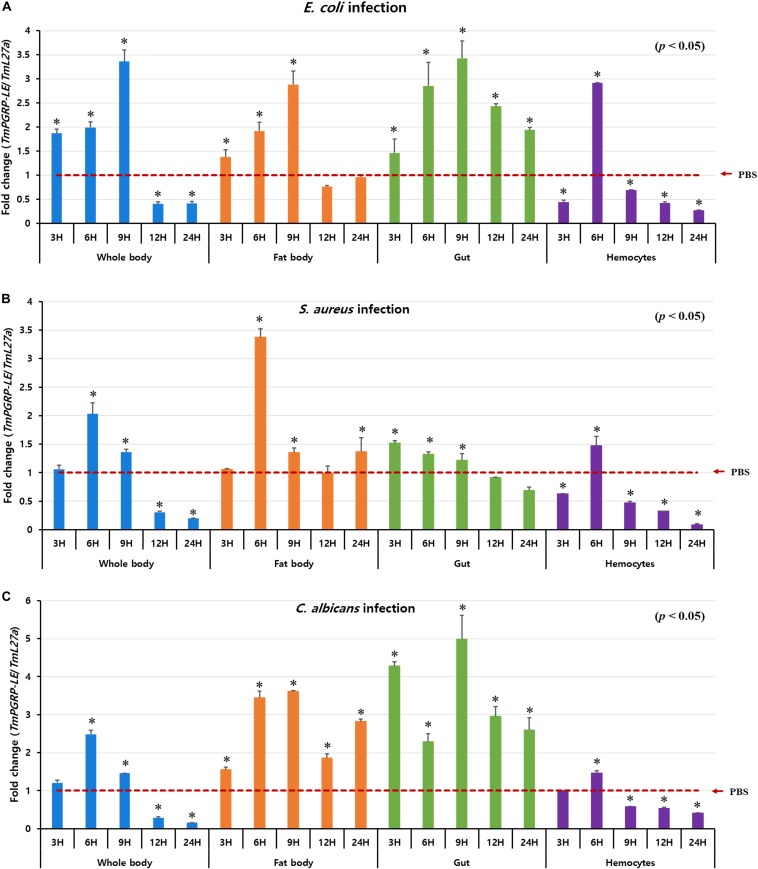
Relative expression profiles of *TmPGRP-LE* in the whole body, fat body, gut, and hemocytes of *T. molitor* young (10th–12th instar) larvae, after experimental challenge with *E. coli* **(A)**, *S. aureus* **(B)**, and *C. albicans* **(C)** (*n* = 20 per treatment group per time point) at 3, 6, 9, 12, and 24 h post-infection were examined by qRT-PCR. *TmPGRP-LE* mRNA levels were normalized against the internal control, *T. molitor 60S ribosomal protein L27a* (*TmL27a*), followed by normalization against the average relative expression of PBS-challenged controls. Each vertical bar represents mean ± SE. Statistical differences are denoted by asterisks (*) (*p* < 0.05).

The *Drosophila* PGRP-LE protein serves as a master bacterial peptidoglycan-sensing molecule in the gut that mediates NF-κB-induced responses to infectious pathogens (Bosco-Drayon et al., 2012). Thus, to elucidate how whole organs and tissues of *T. molitor* (including fat body, gut, and hemocytes) respond to various microbes, we evaluated changes in the transcriptional abundance of *TmPGRP-LE* after challenging hosts with *E. coli*, *S. aureus*, and *C. albicans* at various time points (3, 6, 9, 12, and 24 h post-infection) (Figure 2). In the whole body, fat body, and gut tissues, *E. coli* infection led to a gradual but significant upregulation in transcription of *TmPGRP-LE*, resulting in an up to threefold increase in expression over the PBS-injected controls by 9 h post-infection (*p* < 0.05). Levels of *TmPGRP-LE* in the whole body and fat body increased at early time points (3, 6, and 9 h) but did not persist at 12 and 24 h post-infection. Similarly, *TmPGRP-LE* expression levels were significantly increased in the gut, but there was a slight decrease (*p* < 0.05) in expression after 9 h (Figure 2A). These results indicate that the *T. molitor* larval gut responds to infection by *E. coli* and that the responses involve *TmPGRP-LE*.

As shown in Figure 2B, *S. aureus* challenge primarily induced *TmPGRP-LE* expression at 6 h post-infection in the whole body and fat body, whereas its expression dropped noticeably at later time points. In the gut, *S. aureus* exposure resulted in weak expression of *TmPGRP-LE* and its expression remained mostly unchanged at 3, 6, and 9 h post-infection (*p* < 0.05) (Figure 2B). Similar results were observed for *TmPGRP-LE* transcription following exposure to *C. albicans* as were found after infection with *E. coli*. The fold-increase in *TmPGRP-LE* mRNA levels was highest at 9 h post-infection with *C. albicans* in the fat body and gut in comparison to mock controls (*p* < 0.05) (Figure 2C).

Although microbial infection (*E. coli, S. aureus*, and *C. albicans*) within hemocytes was different in *T. molitor* larvae, the mRNA expression patterns of *TmPGRP-LE* were extremely similar. These results show a significant increase in *TmPGRP-LE* expression 6 h after the onset of infection followed by a dramatic decline in expression during later time points (*p* < 0.05) (Figures 2A–C). In hemocytes, the expression level of *TmPGRP-LE* after *E. coli* infection was more potent than it was after infection with *S. aureus* and *C. albicans* (Figure 2A).

### Effect of *TmPGRP-LE* Silencing on Larval Survival in *T. molitor* After Microbial Insults

**FIGURE 3:**
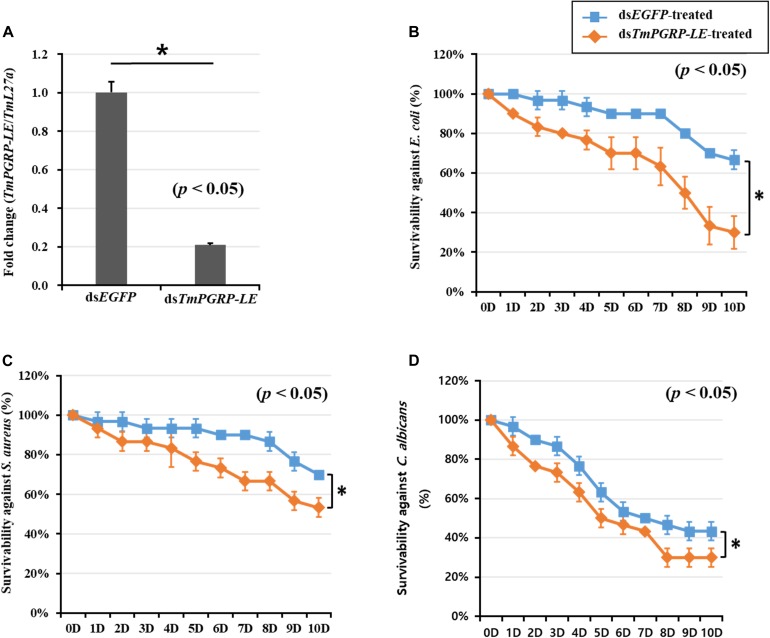
Effect of *TmPGRP-LE* silencing on the larval survival of *T. molitor* (*n* = 10 per treatment group), monitored for 10 days after microbial challenge. Knockdown efficiency of *TmPGRP-LE* in *T. molitor* larvae injected with target gene-specific dsRNA **(A)**. Young larvae (*n* = 3 per group) were injected with 1 μL (1 μg) of *TmPGRP-LE* dsRNA and, after 6 days, the *TmPGRP-LE* transcript was determined by qRT-PCR. Survival of ds*TmPGRP-LE*-injected larvae during immune challenge with *E. coli* **(B)**, *S. aureus* **(C)**, and *C. albicans* **(D)**. Larvae injected with dsRNA targeting *enhanced green fluorescent protein* (ds*EGFP*) was used as a negative control. Results are an average of three independent biological replicates. Asterisks (*) denote significant differences between *EGFP*- and *TmPGRP-LE*-silenced larvae (*p* < 0.05).

In insects, the conserved RNAi pathway has been widely described, and RNAi techniques using dsRNA injection have been applied for gene function studies for over a decade (Brown et al., 1999; Amdam et al., 2003). To assess the response of the *T. molitor* young larvae (10th–12th instar) to ds*TmPGRP-LE* injection compared to ds*EGFP* control injection, the knockdown efficiency was measured by qRT-PCR. The transcription of *TmPGRP-LE* was significantly reduced (80%) 6 days after dsRNA injection (*p* < 0.05) (Figure 3A). As expected, silencing of *TmPGRP-LE* had no effect on PBS-injected larvae mortality (Supplementary Figure S1). Furthermore, in order to determine the necessity of *TmPGRP-LE* in larval survival, we challenged dsRNA-treated larvae with *E. coli*, *S. aureus*, and *C. albicans* and assessed insect mortality over 10 days. The survival rate of *TmPGRP-LE*-silenced larvae that were infected with *E. coli* dropped dramatically to 30% compared to >60% survival of the controls (*p* < 0.05) (Figure 3B). The percent survival resulting from *S. aureus* (50%, Figure 3C) and *C. albicans* (30%, Figure 3D) challenges in the *TmPGRP-LE*-silenced groups were significantly lower than that in the ds*EGFP*-injected controls (*p* < 0.05).

### Role of *TmPGRP-LE* in Induction of *T. molitor* AMP Genes at 24 h Post-microbial Challenge

Among the previously described receptors of the Imd signaling pathway, *Dm*PGRP-LE senses bacteria-derived PGN either independently or synergistically with *Dm*PGRP-LC depending on its localization. It eventually conveys the signal transduction to the NF-κB transcription proteins and in-turn regulates immunity effector genes, i.e., AMPs (Takehana et al., 2004). Considering the significant mortality ratios observed in *TmPGRP-LE*-silenced larvae groups after microbial invasion, we reasoned that these phenotypes could be due to *TmPGRP-LE* knockdown which would subsequently impair the antimicrobial responses. These observations prompted us to investigate the expression levels of 14 AMP genes in larvae with depleted levels of *TmPGRP-LE*. Therefore, we injected 1 μL (1 μg) of ds*TmPGRP-LE* and ds*EGFP* into two sets of *T. molitor* larvae and confirmed the knockdown efficiency (80%) of the target genes after 6 days. Given that immune tissues might be variably tolerant to infection (Neyen et al., 2012), we examined the larval fat body, gut, and hemocytes of ds*TmPGRP-LE-* and ds*EGFP*-treated cohorts 24 h after challenge with *E. coli*, *S. aureus*, and *C. albicans*.

**FIGURE 4:**
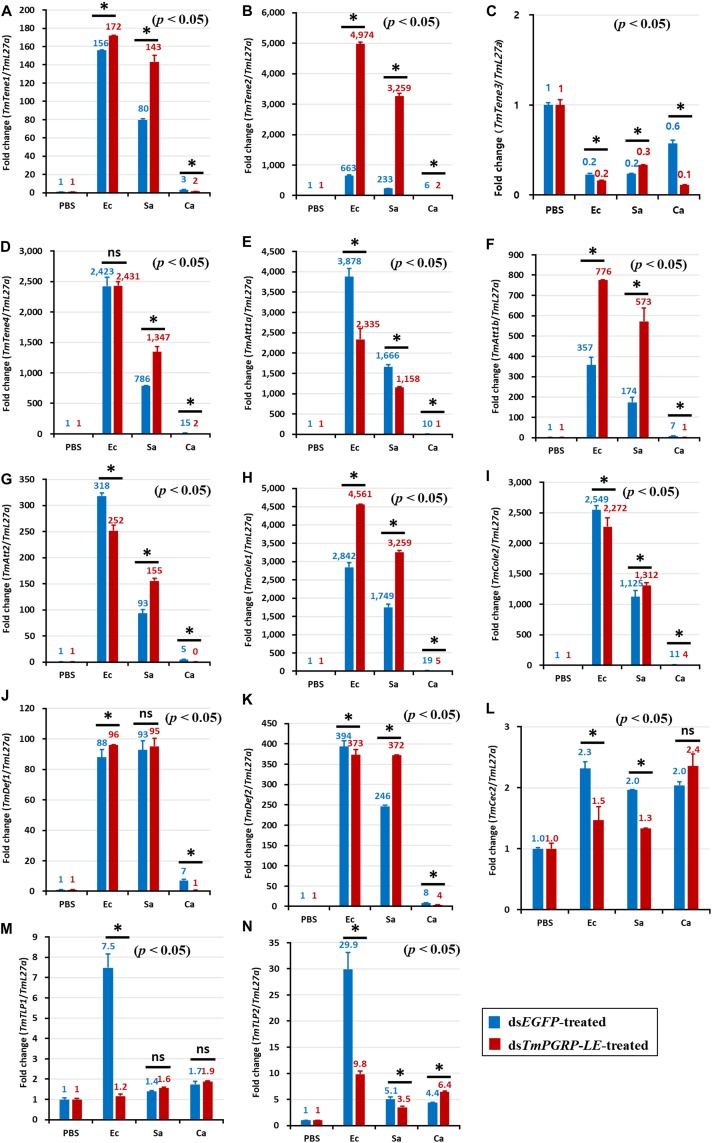
Antimicrobial peptide (AMP) induction profiles in the *TmPGRP-LE-*silenced *T. molitor* larval fat body in response to *E. coli* (Ec), *S. aureus* (Sa), and *C. albicans* (Ca) challenges. Double-stranded RNA specific to *TmPGRP-LE* was injected (1 μg) into young larvae. Six days after dsRNA treatment, *TmPGRP-LE* mRNA levels were reduced by 80% in the ds*TmPGRP-LE*-injected groups compared to ds*EGFP*-injected groups. The larvae were then infected with *E. coli*, *S. aureus*, and *C. albicans* (*n* = 20 per group). The expression levels of 14 AMP genes were evaluated by qRT-PCR: *TmTenecin-1* (*TmTene1*, **A**); *TmTenecin-2* (*TmTene2*, **B**); *TmTenecin-3* (*TmTene3*, **C**); *TmTenecin-4* (*TmTene4*, **D**); *TmAttacin-1a* (*TmAtt1a*, **E**); *TmAttacin-1b* (*TmAtt1b*, **F**); *TmAttacin-2* (*TmAtt2*, **G**); *TmColeptericin-1* (*TmCole1*, **H**); *TmColeptericin-2* (*TmCole2*, **I**); *TmDefensin-1* (*TmDef1*, **J**); *TmDefensin-2* (*TmDef2*, **K**); *TmCecropin-2* (*TmCec2*, **L**); *TmTLP-1* (*TmTLP1*, **M**); and *TmTLP-2* (*TmTLP2*, **N**). *EGFP* dsRNA injection served as a negative control, and the mRNA levels of the respective AMP genes are presented relative to those for *TmL27a* as an internal control. Each vertical bar represents mean ± SE of three independent biological replicates and the numbers above the bars show AMP transcription levels. Significant differences between ds*EGFP*- and ds*TmPGRP-LE*-treated cohorts are shown by asterisks (*) (*p* < 0.05) and ns, not significant.

**FIGURE 5:**
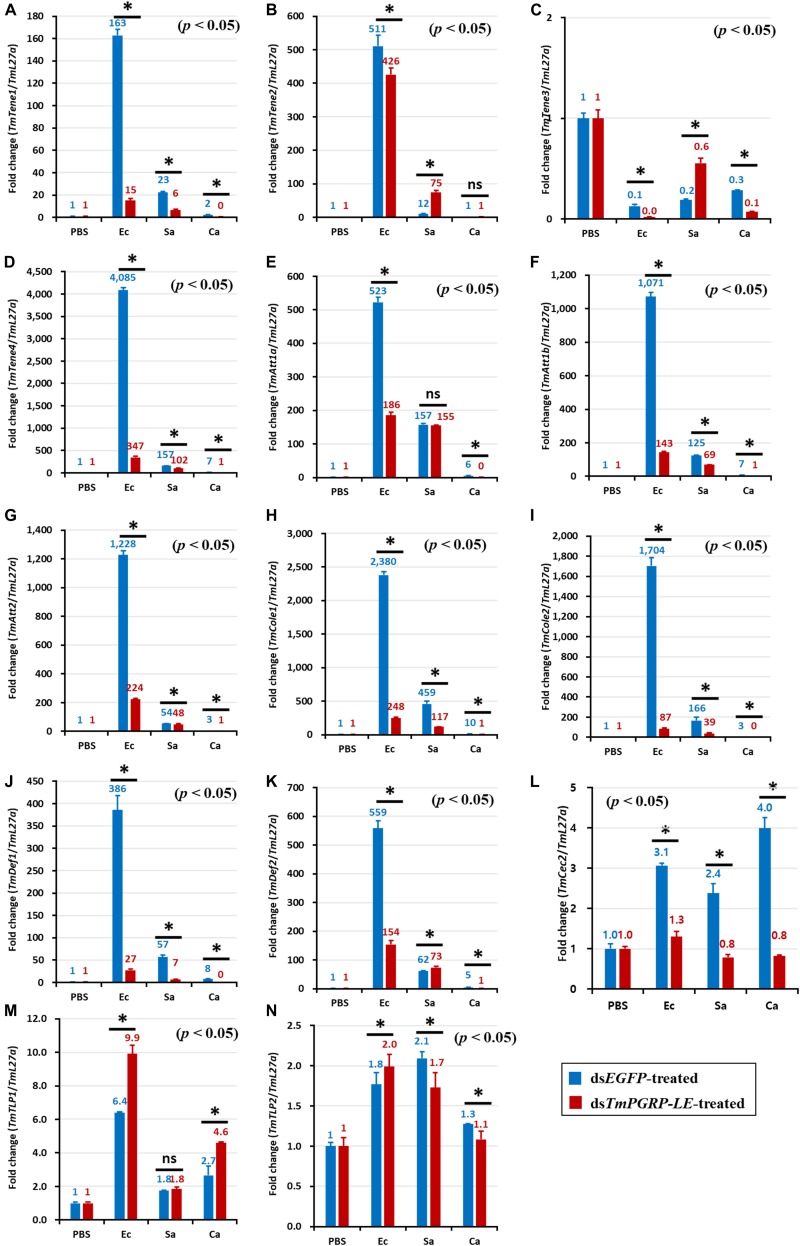
The effect of *TmPGRP-LE* silencing on induction of antimicrobial peptides (AMPs) in the gut of *T. molitor* larvae in response to pathogenic microbial stimuli of *E. coli* (Ec), *S. aureus* (Sa), and *C. albicans* (Ca). Young *T. molitor* larvae were treated with 1 μL (1 μg) of dsRNA of *TmPGRP-LE*. Six days later, the dsRNA-treated larvae were challenged with *E. coli*, *S. aureus*, and *C. albicans* (*n* = 20 per group). The mRNA quantity of 14 AMP genes, namely *TmTenecin-1* (*TmTene1*, **A**), *TmTenecin-2* (*TmTene2*, **B**), *TmTenecin-3* (*TmTene3*, **C**), *TmTenecin-4* (*TmTene4*, **D**), *TmAttacin-1a* (*TmAtt1a*, **E**), *TmAttacin-1b* (*TmAtt1b*, **F**), *TmAttacin-2* (*TmAtt2*, **G**), *TmColeptericin-1* (*TmCole1*, **H**), *TmColeptericin-2* (*TmCole2*, **I**), *TmDefensin-1* (*TmDef1*, **J**), *TmDefensin-2* (*TmDef2*, **K**), *TmCecropin-2* (*TmCec2*, **L**), *TmTLP-1* (*TmTLP1*, **M**), and *TmTLP-2* (*TmTLP2*, **N**), were measured in relation to *L27a* by qRT-PCR 24 h post-infection. The other details were the same as in Figure 4.

In ds*EGFP*-injected controls, *E. coli* infection induced the expression of *TmTene1* (Figures 4A, 5A), *TmTene2* (Figures 4B, 5B), *TmTene4* (Figures 4D, 5D), *TmAtt1a* (Figures 4E, 5E), *TmAtt1b* (Figures 4F, 5F), *TmAtt2* (Figures 4G, 5G), *TmCole1* (Figures 4H, 5H), *TmCole2* (Figures 4I, 5I), *TmDef1* (Figures 4J, 5J), *TmDef2* (Figures 4K, 5K), and *TmCec2* (Figures 4L, 5L) in both the fat body and gut (Figures 4, 5). In ds*TmPGRP-LE*-injected groups, *E. coli*-induced gut expression of all 11 AMP genes was significantly downregulated (Figure 5), while fat body expression of *TmTene1* (Figure 4A), *TmTene2* (Figure 4B), *TmAtt1b* (Figure 4F), *TmCole1* (Figure 4H), and *TmDef1* (Figure 4J) was upregulated.

In the fat body, *C. albicans*-induced expression of almost all AMPs was decreased in *TmPGRP-LE*-silenced larvae with the exception of four AMPs: *TmTene3* (Figure 4C), *TmCec2* (Figure 4L), *TmTLP1* (Figure 4M), and *TmTLP2* (Figure 4N). This suggests that *TmPGRP-LE* is crucial for the production of AMPs after *C. albicans* infection (Figure 4). In contrast, *S. aureus-*dependent expression of AMPs was dramatically increased in *TmPGRP-LE*-silenced larvae with the exception of *TmAtt1a* (Figure 4E), *TmCec2* (Figure 4L), and *TmTLP2* (Figure 4N). Furthermore, *TmPGRP-LE* knockdown did not affect the mRNA levels of *TmDef1* (Figure 4J) and *TmTLP1* (Figure 4M) after *S. aureus* infection and of *TmCec2* (Figure 4L) and *TmTLP1* (Figure 4M) following *C. albicans* challenge.

Notably, AMP genes were upregulated dramatically in the gut of ds*EGFP*-injected larvae infected with *E. coli* (Figure 5). The upregulation of *TmTene1* (Figure 5A), *TmTene2* (Figure 5B), *TmTene4* (Figure 5D), *TmAtt1a* (Figure 5E), *TmAtt1b* (Figure 5F), *TmAtt2* (Figure 5G), *TmCole1* (Figure 5H), *TmCole2* (Figure 5I), *TmDef1* (Figure 5J), and *TmDef2* (Figure 5K) was notably suppressed in ds*TmPGRP-LE*-treated larvae. This result highlights the critical role of *TmPGRP-LE* in the humoral immune response of the gut.

However, in contrast to the strong AMP induction observed in the gut following *E. coli* challenge, AMP expression was comparatively mild in *S. aureus* and *C. albicans* infections.

Consistently, silencing of *PGRP-LE* decreased the expression of *S. aureus*-induced AMP genes, including *TmTene1* (Figure 5A), *TmTene4* (Figure 5D), *TmAtt1b* (Figure 5F), *TmAtt2* (Figure 5G), *TmCole1* (Figure 5H), *TmCole2* (Figure 5I), *TmDef1* (Figure 5J), and *TmCec2* (Figure 5L) in comparison with their levels in ds*EGFP*-injected larvae. Compared with the prominent effect of *TmPGRP-LE* on AMPs expression upon *E. coli* challenge, *TmPGRP-LE* acts as a positive regulator during *S. aureus* infection, where it conveys the signal to produce AMPs. As shown Figure 5, silencing *TmPGRP-LE* had surprising effects on the induction of gut AMPs by *C. albicans*. More precisely, the mRNA levels of 11 AMP genes, namely *TmTene1* (Figure 5A), *TmTene4* (Figure 5D), *TmAtt1a* (Figure 5E), *TmAtt1b* (Figure 5F), *TmAtt2* (Figure 5G), *TmCole1* (Figure 5H), *TmCole2* (Figure 5I), *TmDef1* (Figure 5J), *TmDef2* (Figure 5K), and *TmCec2* (Figure 5L), were significantly decreased.

**FIGURE 6:**
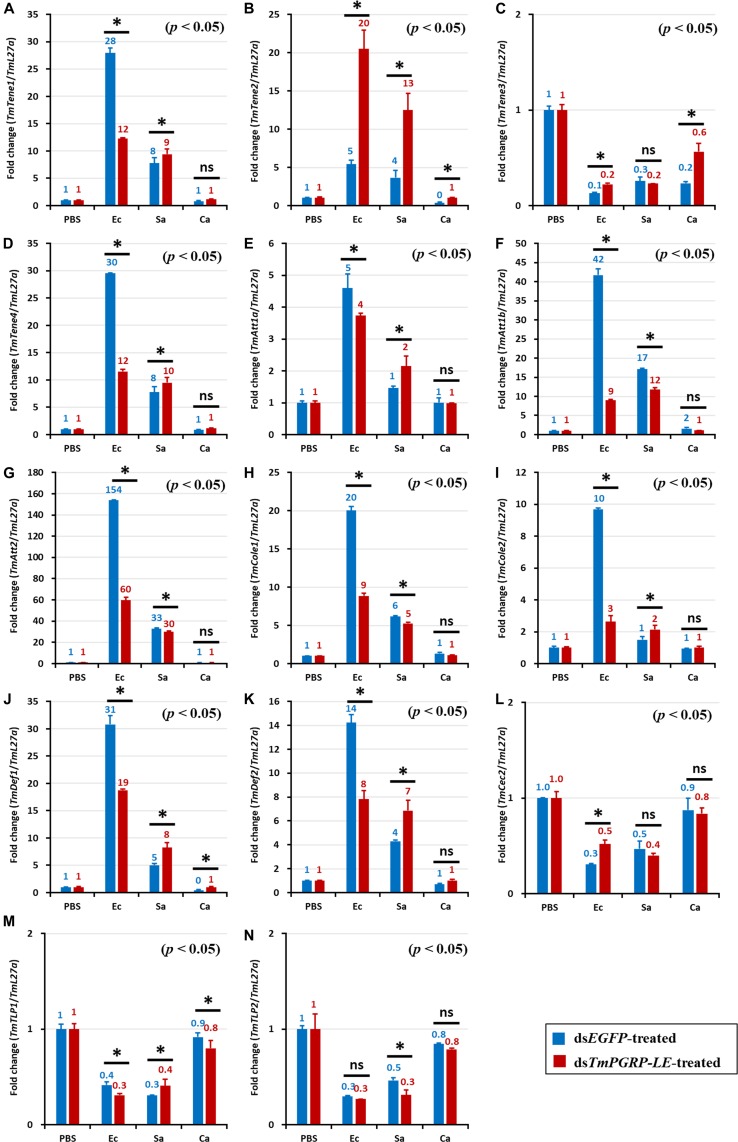
Effect of *TmPGRP-LE* knockdown on induction of microbial AMP genes in the hemocytes of *T. molitor* larvae in response to *E. coli* (Ec), *S. aureus* (Sa), and *C. albicans* (Ca) infections. On the sixth day after ds*TmPGRP-LE* injection (knockdown efficiency of 80%), the larvae were experimentally exposed to *E. coli*, *S. aureus*, or *C. albicans* (*n* = 20 per group). Twenty-four hours post-infection; the larvae were dissected. qRT-PCR was used to evaluate the expression profile of 14 AMP genes: *TmTenecin-1* (*TmTene1*, **A**), *TmTenecin-2* (*TmTene2*, **B**), *TmTenecin-3* (*TmTene3*, **C**), *TmTenecin-4* (*TmTene4*, **D**), *TmAttacin-1a* (*TmAtt1a*, **E**), *TmAttacin-1b* (*TmAtt1b*, **F**), *TmAttacin-2* (*TmAtt2*, **G**), *TmColeptericin-1* (*TmCole1*, **H**), *TmColeptericin-2* (*TmCole2*, **I**), *TmDefensin-1* (*TmDef1*, **J**), *TmDefensin-2* (*TmDef2*, **K**), *TmCecropin-2* (*TmCec2*, **L**), *TmTLP-1* (*TmTLP1*, **M**), and *TmTLP-2* (*TmTLP2*, **N**). The other details were the same as in Figure 4.

The antimicrobial responses to fungal and bacterial challenges in hemocytes of ds*TmPGRP-LE*-treated larvae were weaker compared to the other tissues studied (Figure 6). Similar to fat body and gut responses, when ds*TmPGRP-LE*-injected larvae were challenged with *E. coli*, the transcription levels of *TmTene1* (Figure 6A), *TmTene4* (Figure 6D), *TmAtt1a* (Figure 6E), *TmAtt1b* (Figure 6F), *TmAtt2* (Figure 6G), *TmCole1* (Figure 6H), *TmCole2* (Figure 6I), *TmDef1* (Figure 6J), and *TmDef2* (Figure 6K) were downregulated compared to ds*EGFP*-treated controls. In contrast, however, *TmTene1* (Figure 6A), *TmTene2* (Figure 6B), *TmTene4* (Figure 6D), *TmAtt1a* (Figure 6E), *TmCole2* (Figure 6I), *TmDef1* (Figure 6J), and *TmDef2* (Figure 6K) gene expression following infection with *S. aureus* remained inducible in ds*TmPGRP-LE*-injected larvae, suggesting that *TmPGRP-LE* acts as a negative signal transducer in hemocytes following *S. aureus* challenge. Note that levels of expression of AMP genes in larval hemocytes after challenge with *C. albicans* were not significantly different in *TmPGRP-LE*-silenced larvae compared to ds*EGFP* controls (Figure 6).

Altogether, these results indicate that reducing *TmPGRP-LE* levels in the fat body, gut, and hemocytes led to a significant downregulation of AMP genes after *E. coli* infection. Similarly, *TmPGRP-LE* depletion was sufficient to suppress the expression of several AMP genes in the fat body and gut following *C. albicans* challenge, highlighting the critical role of *TmPGRP-LE* as a receptor of the NF-κB signaling pathway. In contrast to the responses to *E. coli* and *C. albicans* infection, no drastic reduction in the transcription of AMP genes was observed during *S. aureus* infections in the fat body of *TmPGRP-LE*-silenced larvae.

### Effect of *TmPGRP-LE* RNAi on Expression of *T. molitor* NF-κB Genes After Pathogenic Microbial Stimuli

Previous studies in insect immunity have revealed that different transcription factors regulate the Toll and Imd immunity pathways. Relish and Dorsal, key downstream proteins in the Imd and Toll pathways, respectively, from dimers to participate in the precise control of the production of AMPs (Sagisaka et al., 2004; Shin et al., 2005; Schlüns and Crozier, 2007; Tanaka et al., 2007; Oeckinghaus and Ghosh, 2009; Keshavarz et al., 2019). We wanted to find out whether the reduction of *TmPGRP-*LE, which is a sensor of PGN (Tindwa et al., 2013), would have an effect on the expression levels of *T. molitor* transcription proteins. To address this question, we assessed the mRNA expression of *TmRelish*, *TmDorsal X1* isoform (*TmDorX1*), and *TmDorsal X2* isoform (*TmDorX2)* in the fat body, gut, and hemocytes of *TmPGRP-LE-*silenced larvae following infection with *E. coli*, *S. aureus*, and *C. albicans*.

**FIGURE 7:**
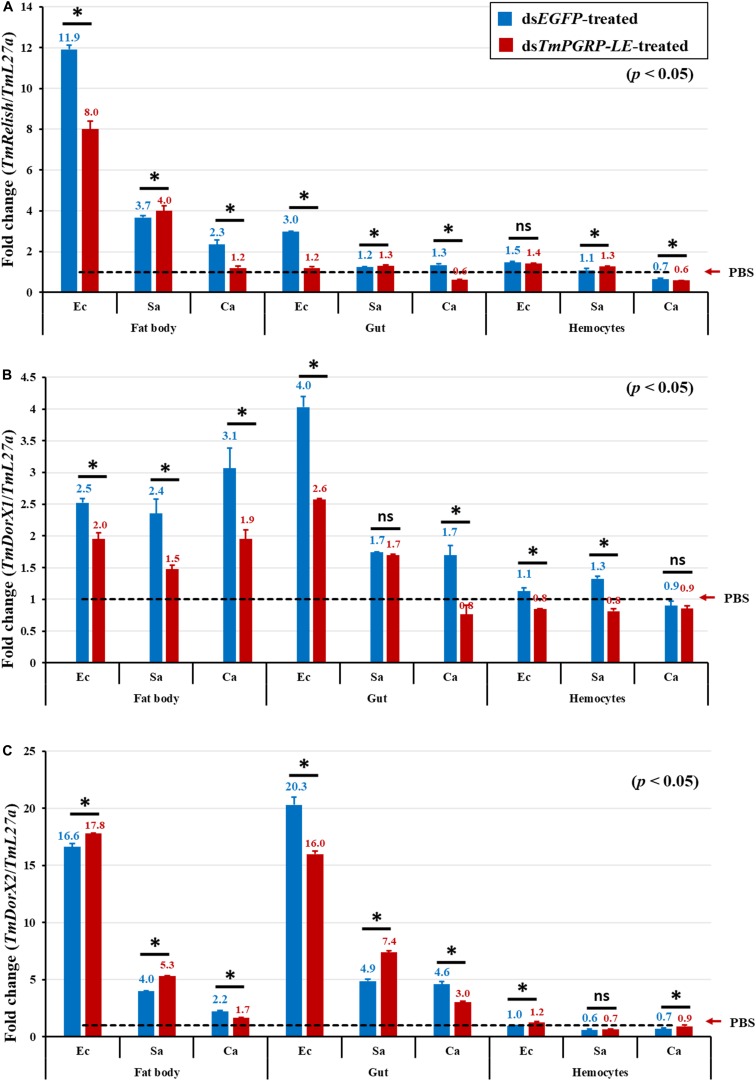
Transcriptional activation of different NF-κB genes in the fat body, hemocytes, and gut of *TmPGRP-LE* dsRNA-injected *T. molitor* larvae after inoculation with *E. coli* (Ec), *S. aureus* (Sa), and *C. albicans* (Ca) (*n* = 20 per group). mRNA quantities of *TmRelish* **(A)**, *TmDorX1* **(B)**, and *TmDorX2* **(C)** in *TmPGRP-LE* knockdown larvae were measured relative to those for *L27a* at 24 h post-challenge by qRT-PCR. *EGFP* RNAi was used as a negative control. Bars represent mean ± SE of three independent experiments and the numbers above the bars indicate the transcription levels of NF-κB genes. Significant differences between ds*EGFP*- and ds*TmPGRP-LE*-treated groups are presented by asterisks (*) (*p* < 0.05) and ns, not significant.

After *E. coli* and *C. albicans* infections, depletion of *TmPGRP-LE* appeared to have a significant effect in downregulating *TmRelish* and *TmDorX1* transcription in the larval fat body and gut (Figures 7A, B). *TmPGRP-LE* knockdown larvae showed slightly decreased *TmDorX1* expression in the fat body and hemocytes after *S. aureus* infection (Figure 7B).

In ds*TmPGRP-LE*-injected larvae, expression of *TmDorX2* was significantly decreased in both the fat body and gut following *C. albicans* challenge (Figure 7C). In response to *E. coli* infection, there was a moderate decline in the expression of *TmDorX2* in the gut (Figure 7C) and of *TmDorX1* in hemocytes (Figure 7B).

Collectively, all NF-κB genes were significantly downregulated in the larval gut of *TmPGRP-LE* knockdown groups after infection with either *E. coli* or *C. albicans*. Of note, a similar result was observed in the fat body of ds*TmPGRP-LE*-injected larvae following *C. albicans* challenge. It is plausible that the Toll and Imd pathways synergistically activate through *TmPGRP-LE* (Tanji et al., 2007). This conclusion is in agreement with our findings regarding the expression of AMP genes in the fat body and gut.

## Discussion

The recognition of foreign microbes is a crucial aspect of the host defense mechanism that relies on the activation of PGRPs as central sensors and regulators of innate immune response. *Dm*PGRP-LC is a transmembrane protein in the Imd pathway, which is fundamental to the production of an array of potent AMPs following *E. coli* challenge (Rämet et al., 2002). It is not, however, the only upstream factor that activates the Imd pathway (Gottar et al., 2002). The multifunctional protein PGRP-LE, a constitutive hemolymph protein, triggers both the activation of the prophenoloxidase (pro-PO) cascade and the Imd pathway (Takehana et al., 2002). Importantly, *DmPGRP-LE* is expressed in many cells and tissues, including the fat body, hemocytes, hemolymph, gut, trachea, Malpighian tubules, and cuticle (Takehana et al., 2002, 2004; Kaneko et al., 2006). In this study, we found that *TmPGRP-LE* was expressed in the integument, fat body, hemocytes, gut, and Malpighian tubules, which is in agreement with the results of previous studies. Interestingly, we observed that *TmPGRP-LE* expression was comparatively higher in the gut and hemocytes of *T. molitor* both larvae and adults. The results of our study, along with previously published results (Bosco-Drayon et al., 2012; Neyen et al., 2012), may indicate that PGRP-LE is necessary and sufficient to react promptly to PGN in both tissues. Additionally, in the case of hemocytes, considerable expression of *TmPGRP-LE* is related to the activation of the proPO cascade as a secondary humoral response (Takehana et al., 2002). Our results revealed a *TmPGRP-LE*-mediated induction of the gut immune response following *E. coli* infection. Likewise, *T. castaneum* and *Armigeres subalbatus* showed modest expression of *PGRP-LE* after *E. coli* infection (Wang and Beerntsen, 2013; Koyama et al., 2015).

In *Drosophila*, a combination of multiple receptors, namely PGRP-SA, PGRP-SD, and gram-negative binding protein 1 (GNBP1), is required to trigger an adequate immune response against gram-positive bacteria (*S. aureus*) (Bischoff et al., 2004). However, further investigation underlined the main role of PGRP-SD in presenting the PGN of gram-negative bacteria to PGRP-LC, which in-turn activates the Imd pathway (Iatsenko et al., 2016). The interaction between PGRP-SD and PGRP-LE remains to be elucidated. Similar to the *Drosophila* model, the *Tenebrio* PGRP-SA and GNBP1 complex is critical for the activation of the Toll and proPO pathways by Lys-type PGN of *S. aureus* (Park et al., 2007). Here we reported that *TmPGRP-LE* was slightly, but significantly, expressed following *S. aureus* challenge. Unexpectedly, we found that *TmPGRP-LE* is markedly induced in *C. albicans*-infected larvae in both the fat body and gut. Previous genomic analysis of *T. castaneum* revealed a poor activation in *TcPGRP-LE* expression in *C. albicans*-challenged beetles (Zou et al., 2007).

*T. molitor* larvae with decreased levels of *TmPGRP-LE* (silenced with RNAi) have reduced viability following infection. We propose that the most plausible cause of *TmPGRP-LE*-silenced larval death is related to the transcription of AMP genes, which are regulated by two intracellular signaling pathways: the Toll and the Imd pathways. Under this framework, if expression levels of AMPs decline as a result of *TmPGRP-LE* depletion, *TmPGRP-LE* can be considered a positive regulator of AMPs in larvae, and *TmPGRP-LE*-silenced larvae are more susceptible to microbial infections.

In a previous study, expression levels of *TmTene1*, *TmTene2*, *TmTene4*, *TmAtt1a*, *TmAtt1b*, *TmAtt2*, *TmCole1*, *TmCole2*, and *TmDef2* were significantly reduced in *E. coli*-challenged *T. molitor* following silencing of immune deficiency (*Tm*IMD) expression (Jo et al., 2019). These findings are consistent with our own, in which the expression levels of these genes were downregulated in the gut of *E. coli*-challenged *T. molitor* larvae following silencing of *TmPGRP-LE*. This raises the question of whether expression of NF-κB genes are affected by *TmPGRP-LE* RNAi in the gut. In this manuscript, we report that silencing of *TmPGRP-LE* reduced the expression levels of larval gut *TmRelish*, *TmDorX1*, and *TmDorX2* after *E. coli* challenge. This is consistent with our recent report demonstrating that all the aforementioned AMPs were positively regulated by *Tm*DorX2 (Keshavarz et al., 2019) and *Tm*Relish (Keshavarz et al., 2020). These results leave open the possibility that detection of invading *E. coli* by *TmPGRP-LE* in the gut of *T. molitor* larvae results in signal transduction to both *Tm*DorX2 and *Tm*Relish. Although, more work is needed to examine whether translocation of *Tm*DorX2 and *Tm*Relish occurs in ds*TmPGRP-LE* larvae. Furthermore, following *E. coli* infection, the expression of seven AMP genes, namely *TmTene1*, *TmTene4*, *TmAtt1b*, *TmAtt2*, *TmCole1*, *TmCole2*, and *TmDef2*, showed a dramatic reduction in the hemocytes of the ds*TmPGRP-LE*-injected group. We also know that the same AMPs were downregulated in the ds*TmIMD*, ds*TmRelish*, and ds*TmDorX2*-injected cohorts (Jo et al., 2019; Keshavarz et al., 2019). Moreover, in the fat body of ds*TmPGRP-LE*-injected insects, *TmAtt1a*, *TmCole2*, *TmDef2*, and *TmCec2* were significantly suppressed following exposure to *E. coli*. Similarly, depletion of *TmIMD*, *TmRelish*, and *TmDorX2* led to a reduction in the same AMPs in the larval fat body after *E. coli* infection (Jo et al., 2019; Keshavarz et al., 2019). Mortality assays and AMP expression analyses from our present work and those of others suggest that, in comparison to hemocytes and the fat body, the *T. molitor* gut plays a pivotal role in the response to *E. coli* infection via activation of the Imd pathway. Additionally, these findings showed that there was robust *TmPGRP-LE*-dependent AMPs induction following *E. coli* in the larval gut, while there was slight *TmPGRP-LE*-dependent transcription of AMP genes following *S. aureus* and *C. albicans*. Collectively, the presently-identified signaling components of the Imd pathway (comprising *TmPGRP-LE*, *TmIMD*, and *TmRelish*) positively regulate *TmAtt1a*, *TmCole2*, and *TmDef2* gene expression following *E. coli* infection in immune-related tissues (fat body, gut, and hemocytes).

*Drosophila* gut immune defense mechanisms function independently from Imd-induced AMPs following infection by the gram-positive bacteria, *S. aureus* (Hori et al., 2018). As opposed to *Drosophila*, *S. aureus*-infected *T. molitor* larvae exhibited increased mortality following *TmPGRP-LE* silencing, and the transcription of nine AMPs decreased in *TmPGRP-LE* knockdown larval gut. It should also be mentioned that *TmRelish* is a positive regulator of *TmTene1*, *TmTene4*, *TmAtt1b*, *TmAtt2*, *TmCole1*, and *TmCole2* in the gut of *S. aureus*-infected larvae (Keshavarz et al., 2020) suggesting that the Imd pathway is critical for combatting infection of the larval gut infected with *S. aureus* via AMP production. We further show that the involvement of *TmPGRP-LE* in the control of the Imd pathway is limited to only some AMPs in the fat body and hemocytes of *S. aureus*-challenged larvae. *TmAttacin-2*, an anti-gram positive bacterial protein (Jo et al., 2018), is regulated by *TmPGRP-LE* in the fat body, gut, and hemocytes during *S. aureus* infection.

While there is a scarcity of information available regarding the recognition of fungi by PGRP-LE in insects, our current study suggests that *C. albicans* is sensed in the gut and fat body by *TmPGRP-LE*, which leads to the expression of immune-related AMP-coding genes. We observed a notable effect of *TmPGRP-LE* silencing on the mRNA expression of transcription proteins for both Imd (*TmRelish*) and Toll (*TmDorX1* and *2*) signaling pathways in response to *C. albicans* infection in the fat body and gut. In this context, following *C. albicans* challenge, downregulation of *Tm*PGRP-LE*-*induced genes (i.e., AMPs) is mediated by *TmDorX2* in the gut (Keshavarz et al., 2019), whereas the AMP transcription levels are mainly regulated by *TmRelish* in the fat body (Keshavarz et al., 2020).

**FIGURE 8:**
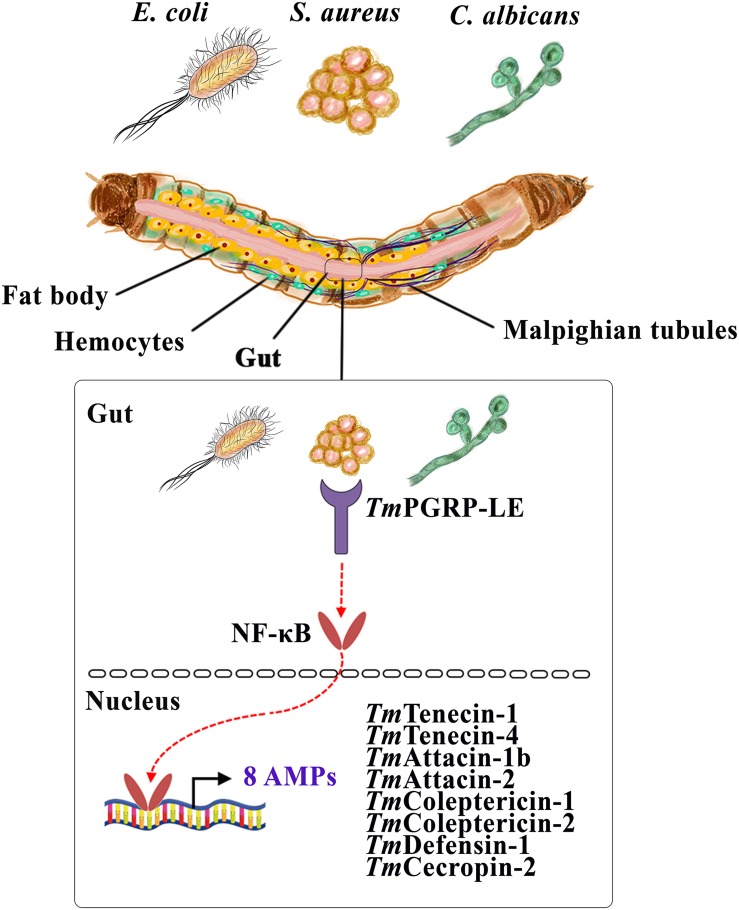
A schematic representation of the role of a central sensor of intracellular immune pathways, *TmPGRP-LE*, in regulating antimicrobial peptides (AMPs) in the larval gut against *E. coli*, *S. aureus*, and *C. albicans*.

Finally, this recent finding uncovers the pivotal role of *TmPGRP-LE* in the immune response of the gut by regulating the production of eight AMP genes, namely *TmTene1*, *TmTene4*, *TmAtt1b*, *TmAtt2*, *TmCole1*, *TmCole2*, *TmDef1*, and *TmCec2*, following infection by *E. coli*, *S. aureus*, and *C. albicans* (Figure 8). Our study provides valuable information about the critical involvement of *TmPGRP-LE* in the regulation of AMP genes in response to microbial infections. More work needs to be done in order to elucidate the cooperation between *TmPGRP-LE* and *T. molitor* NF-κB genes using double-knockdown and immunocytochemistry methods.

## Conclusion

Previous analysis of *T. molitor* PGRP-LE provides insight into the functional role of this non-catalytic PGRP in detecting DAP-type PGN and inducing autophagy, which is the basis of *T. molitor* response to *L. monocytogenes* (Tindwa et al., 2013). However, further studies were needed to explore the exact role of *TmPGRP-LE* in response to gram-negative and gram-positive bacteria and fungi in other innate immune signaling pathways. In this study, we indicate that *TmPGRP-LE* in the *T. molitor* gut induces 10 AMP genes in response to *E. coli* infection. Moreover, *S. aureus*- and *C. albicans*-dependent induction of 9 and 11 AMPs, respectively, are regulated by *TmPGRP-LE* in the larval gut. In conclusion, *TmPGRP-LE* is required for the detection of gram-negative bacteria (*E. coli*) in the gut of *T. molitor*, which subsequently transduces the signal to NF-κB transcription proteins to induce AMPs.

## Data Availability Statement

The datasets generated for this study are available on request to the corresponding author.

## Author Contributions

YH and YJ conceived and designed the experiments and revised the manuscript. MK and TE performed the experiments. MK analyzed the data and wrote the manuscript. YH procured reagents, materials, and analysis tools.

## Conflict of Interest

The authors declare that the research was conducted in the absence of any commercial or financial relationships that could be construed as a potential conflict of interest.

## Footnotes

1 http://www.bioinformatics.nl/cgi-bin/primer3plus/primer3plus.cgi

2 http://www.real-statistics.com/survival-analysis/kaplan-meier-procedure/real-statistics-kaplan-meier/
