# Supplementary material for: Tenebrio molitor PGRP-LE Plays a Critical Role in Gut Antimicrobial Peptide Production in Response to Escherichia coli
Source: Front Physiol. 2020 Apr 15;11:320. doi: 10.3389/fphys.2020.00320 (PMC7179671; doi:10.3389/fphys.2020.00320)
Supplement: Supplementary file 1 [file Table_1.DOCX]

Supplementary Material

# Supplementary Figure


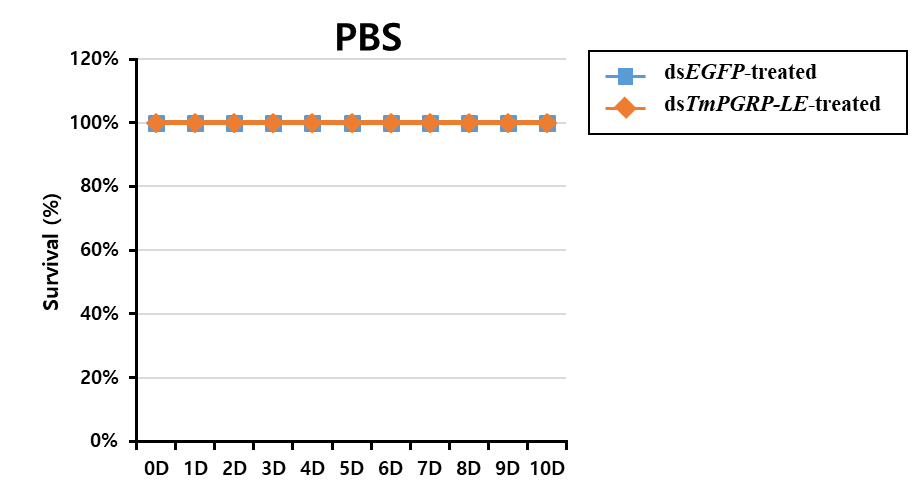


**Supplementary Figure 1.** Effect of *TmPGRP-LE* silencing on the larval survival of *T. molitor* (n = 10 per treatment group), monitored for ten days. Survival of ds*TmPGRP-LE*-injected larvae after PBS (control) injection. Larvae injected with dsRNA targeting *enhanced green fluorescent protein* (ds*EGFP*) was used as a negative control. Results are an average of three independent biological replicates.
